# Spatiotemporal association of DNAJB13 with the annulus during mouse sperm flagellum development

**DOI:** 10.1186/1471-213X-9-23

**Published:** 2009-03-19

**Authors:** Jikui Guan, Makoto Kinoshita, Li Yuan

**Affiliations:** 1 Department of Cell and Molecular Biology, Karolinska Institutet, SE-171 77 Stockholm, Sweden; 2 Biochemistry and Cell Biology Unit, Kyoto University Graduate School of Medicine, Yoshida Konoe, Sakyo, Kyoto 606-8501, Japan

## Abstract

**Background:**

The sperm annulus is a septin-based fibrous ring structure connecting the midpiece and the principal piece of the mammalian sperm flagellum. Although ultrastructural abnormalities and functional importance of the annulus have been addressed in *Sept4*-null mutant mice and a subset of human patients with asthenospermia syndrome, little is known about how the structure is assembled and positioned to the midpiece-principal piece junction during mammalian sperm flagellum development.

**Results:**

By performing immunofluorescence and biochemical approaches with antibodies against DNAJB13 and an annulus constituent SEPT4, we report here a spatiotemporal association of DNAJB13 with sperm annulus during mouse sperm flagellum development. DNAJB13 co-localized with SEPT4 to the annulus, and both were first able to be detected in step 9 spermatids. As spermiogenesis proceeded, the annular DNAJB13 immunosignal increased until the annulus reached the midpiece-principal piece junction, and then gradually disappeared from it in late spermiogenesis. In contrast, the SEPT4 immunosignal was relatively unaltered, and still present on annulus of mature spermatozoa. In *Sept4*-null mouse spermatids lacking the annulus structure, the annulus-like DNAJB13 immunosignal was still able to be detected, albeit weaker, at the neck region of the flagella. In vitro DNAJB13 was co-localized and interacted with SEPT4 directly.

**Conclusion:**

The direct interaction of DNAJB13 with SEPT4 in vitro and its spatiotemporal association with the annulus during sperm flagellum development, and even its annulus-like appearance in the annulus-deficient spermatids, suggest that DNAJB13 may be involved in assembling the annulus structure and positioning it towards the midpiece-principal piece junction.

## Background

The sperm annulus is a ring-like structure existing in all mammalian spermatozoa. This structure was identified 100 years ago and formerly called "Jensen's ring". At low magnification, the annulus appears dense and homogeneous, but at high magnification it shows to be composed of closely packed filamentous subunits oriented circumferentially [1].

During early spermiogenesis, a flagellar axoneme forms beneath the cell surface from one of the two centrioles, and rapidly protudes from the cell. The centriole pair forming the axoneme then move towards the nucleus and finally impacts it, which causes the cell membrane adherent to the centriole to become infolded [2]. As early as in this stage the annulus or the anlage of the annulus can be seen encircling the axoneme at the distal end of the basal body by electron microscopy [3,4]. During the late stage of sperm flagellum development, the annulus slips towards a more distal position, and the mitochondria begin to affix to the flagellum. In a mature spermatozoon the annulus is located between the midpiece and the principal piece of the flagellum, and beneath the plasma membrane, firmly connecting them together [1,3,5-7].

Recent researches show that the sperm annulus is a septin-based structure composed of several septins, such as SEPT1, 4, 6, 7 and 12 [8-10]. Septins, conserved from yeast to *Drosophila *to humans, are polymerizing GTPases that can form hetero-oligomeric filaments required for cytokinesis and other cellular processes [11,12]. The functional importance of septins in sperm annulus formation is illustrated by the *Sept4*-deficient male mice. *Sept4*-deficient male mice are sterile due to defective morphology and motility of the sperm flagella. Mutant sperm lacking the annulus often show bent-back tail morphology at the midpiece-principal piece junction. Therefore, cortical organization of the annulus based on circular assembly of septins is essential for the structural and mechanical integrity of mammalian spermatozoa [8,9]. To date, however, little is known about how the annulus/septin ring is assembled and positioned to the midpiece-principal piece junction during spermiogenesis. In vivo, mammalian septins may need extra factors or adaptors to organize higher-order structures, albeit their self-assembly into filamentous rings in vitro [13]. Thus, the present study intends to examine the involvement of DNAJB13, a type II heat shock protein 40 (HSP40), in annulus assembly and positioning during mammalian sperm flagellum development.

HSP40s generally function as cochaperones in the HSP70 chaperone systems that are involved in many processes in cells, such as folding of newly synthesized proteins, refolding of misfolded proteins, translocation of proteins into organelles, assembly and disassembly of protein complexes, and protein degradation [14-16]. We have previously shown that DNAJB13 is a component of mouse sperm axoneme [17]. Here we report that DNAJB13 is associated with the annulus spatiotemporally during sperm flagellum development in wild type mice, and even present temporarily in the annulus-deficient *Sept4*-null spermatids taking on an annulus-like pattern. In vitro DNAJB13 interacts with SEPT4 directly. Altogether, our data suggest that DNAJB13 may assist in assembling the annulus and positioning it toward the midpiece-principal piece junction during sperm flagellum development.

## Results and discussion

### DNAJB13 localized to sperm annulus transiently during mouse spermiogenesis

**Figure 1:**
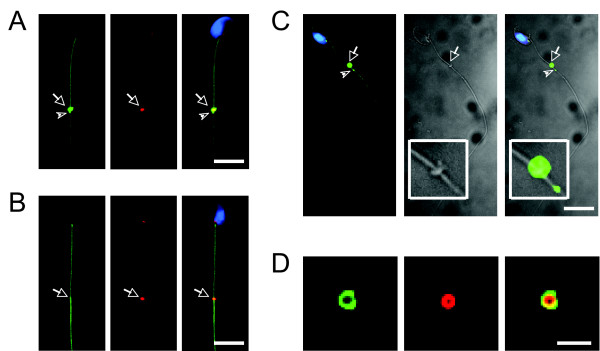
**Transient localization of DNAJB13 to sperm annulus**. In addition to the axonemal localization, DNAJB13 (green) was also co-localized with an annulus constituent SEPT4 (red) to the sperm annulus (arrows) in developing spermatids (A), but not in mature spermatozoa (B). The phase contrast images of a spermatid further showed that the localization of DNAJB13 to the annulus (see insets in the phase contrast images) (C). Note the DNAJB13 antibody also labeled a small dot (arrowheads) below the annulus (A, C). The sperm nuclei were stained with DAPI (blue). The scale bar represents 10 μm; (D) Images of an annulus released from a damaged spermatid. The scale bar represents 2 μm.

With the specific antibody against mouse DNAJB13, we have demonstrated that DNAJB13 is an axonemal component of flagella in mature spermatozoa [17]. Surprisingly, when performing immunofluorescence microscopy on mouse testicular germ cells, we found that this antibody also labeled a ring-like structure in certain spermatids in addition to the axoneme. It is known that three kinds of ring-like structures form during mammalian spermatogenesis: intercellular bridges, the perinuclear ring of the manchette, and the annulus [18]. The ring-like structure labelled by this antibody on flagella prompted us to investigate whether or not DNAJB13 localized to sperm annulus by co-staining with anti-SEPT4 antibody. SEPT4, an annulus constituent, has been used to indicate the sperm annulus [8-10]. We then found that DNAJB13 was co-localized with SEPT4, but only in developing spermatids (Figure 1A; additional file 1), not in mature epididymal spermatozoa (Figure 1B). We also employed phase contrast microscopy to clearly demonstrate the sperm annulus (Figure 1C), which was stained with DNAJB13 antibody in parallel, showing that DNAJB13 indeed localized to the sperm annulus.

Due to the difference in signal intensity and angles of view, the annular DNAJB13 staining exhibited three distinct patterns. In most cases, it showed a bar-like pattern (Figure 1A; figure 3; additional file 2B); but the ring-like pattern (Figure 1D; additional file 1; additional file 2C) was obvious when detached from flagella; it also displayed a two-dot pattern when the signal intensity was low, especially at the beginning of the annulus formation (Additional file 2A). In addition to its annulus location, DNAJB13 staining on the same testicular cell preparation uncovered a discernible pattern. In a subset of spermatids, a small dot labelled with the antibody was kept apart from the annulus staining toward the distal end of the sperm flagellum (Figures 1A, C). During sample preparation, some spermatids were damaged and their annuli were released. In this case, DNAJB13 was still co-localized with SEPT4 on the ring-like structure (Figure 1D), indicating that there may be a strong physical interaction between them.

### Spatiotemporal progression of annular DNAJB13 during spermiogenesis

Intrigued by our observation above, we employed immunofluorescence microscopy to follow the spatiotemporal progression of annular DNAJB13 with the annulus during mouse spermiogenesis in detail. In order to define each step in spermiogenesis, we combined the antibody staining with DAPI (nuclear staining) and MitoTracker (mitochondrial marker). Because it is difficult for us to demonstrate 4 colors in one sample, we stained the testicular germ cells with the SEPT4 antibody and the DNAJB13 antibody separately.

**Figure 2:**
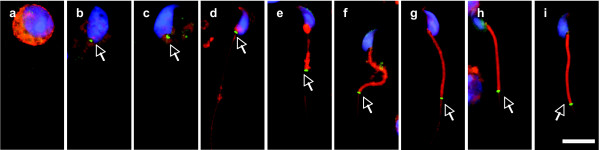
**Spatiotemporal progression of annulus during mouse spermiogenesis**. The annulus (arrows) indicated by the SEPT4 antibody was first seen at the neck region of step 9 spermatids (b), not in step 8 spermatids (a). It stayed there until early step 15 (c-d), and after that it migrated to its distal position followed by the formation of mitochondrial sheath (e-h). It still existed in the mature spermatiozoon coonecting the midpiece and the principal piece of the tail (i). Green, SEPT4; Red, MitoTracker; Blue, DAPI. The scale bar represents 10 μm.

The annulus indicated by the SEPT4 immunofluorescent signal first appeared in step 9 spermatids (Figure 2b), not in step 8 spermatids (Figure 2a). It kept staying at the neck region until the spermatids developed to early step 15 (Figures 2c, d), and after that it slipped down to its distal position followed by the formation of the mitochondrial sheath (Figures 2e–h). Of note, SEPT4 was still present at the midpiece-principal piece junction of mature spermatozoa (Figure 2i). It has been shown by electron microscopy that the annulus can be detected shortly after the flagellum formation and migration toward the nucleus in the early stage of cat spermiogenesis [3]. In Chinese hamster spermiogenesis, the anlage of the annulus is first detected in spermatids in which the centriolar pair forming the flagellum has reached and impacted the nucleus [4]. Correspondingly, the flagellum forms in step 2–3 spermatids, then migrates inwards and finally reaches and indents the nucleus in step 6 spermatids in both rat and mouse spermiogenesis [2]. However, with immunofluorescence microscopy, we were unable to detect the annulus structure even in step 8 spermatids, probably due to the sensitivity difference between the two methodologies, or the developmental difference among different species, or because the annulus prior to step 9 is not septin-based. Indeed, early ultrastructural studies in rat and mouse spermatids show that the annulus cannot be clearly seen until step 9 [2,19], and stays at the neck region from step 9 to early step 15 [2], consistent with our present findings.

**Figure 3:**
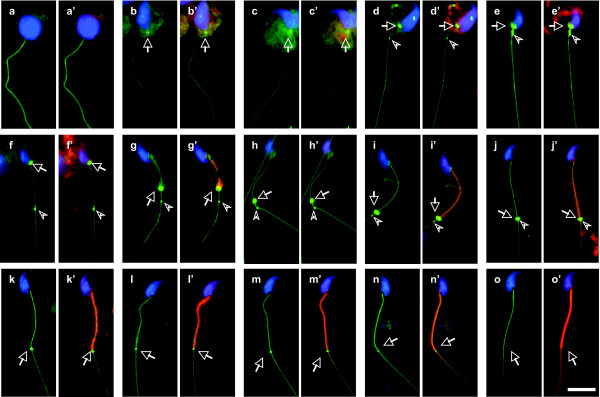
**Spatiotemporal progression of annular DNAJB13 during mouse spermiogenesis**. The annular DNAJB13 staining (green, arrows) was first seen at the neck region of step 9 spermatids (b), not in step 8 spermatids (a), and the staining intensity increased until the annulus slipped down to its distal position at step 15 (c-i). With the formation and maturation of the mitochondrial sheath (red), the signal of annular DNAJB13 decreased gradually from middle step 15 to step 16 (j-n) and disappeared totally in mature spermatozoa (o). Note the DNAJB13 antibody also labeled a dot (arrowheads) below the annulus. This dot was first seen around step 13–14 (d) and migrated towards the midpiece-principal piece junction along the flagellum (e-j). The annulus followed and finally merged with the dot at the junction (j). The sperm nuclei were stained with DAPI (blue), while the mitochondria were stained with MitoTracker. (a-o) Merged images of DNAJB13 with DAPI; (a'-o') Merged images of DNAJB13, MitoTracker and DAPI. The scale bar represents 10 μm.

Coincident with the appearance of annulus demonstrated by the SEPT4 antibody, the annulus-like DNAJB13 immunofluorescence signal was also first seen in step 9 spermatids (Figure 3b), not in earlier step spermatids (Figure 3a). The signal intensity of annular DNAJB13 increased as the spermiogenesis proceeded (Figures 3c–i). It peaked when the annulus migrated down to its most distal position, and afterwards decreased gradually with the formation and maturation of mitochondrial sheath (Figures 3j–n). In contrast to SEPT4 staining (Figure 2i), DNAJB13 was absent at the midpiece-principal piece junction of mature spermatozoa (Figure 3o). The nuclear staining (blue) and mitochondrial staining (red) used to define steps of spermiogenesis were merged with the DNAJB13 staining to clearly show the whole progression (Figures 3a'–o'). As shown in Figure 1A, figures 3d, e, f, g, h, i and 3j detailed the migrating progress of both DNAJB13-labeled dot and annulus towards the midpiece-principal piece junction on the tail. The DNAJB13 dot appeared around step 13–14 (Figure 3d), a time point slightly before annulus migration (Figures 3e), advanced toward the midpiece-principal piece junction (Figures 3f, g), and then stayed there (Figures 3h–j). The DNAJB13-labeled annulus followed the DNAJB13 dot downwards (Figures 3g–i), and eventually merged with it at the midpiece-principal piece junction (Figure 3j). These data indicate that the annular DNAJB13 and the DNAJB13 dot may be involved in positioning of the annulus.

### Localization of DNAJB13 in Sept4-null spermatids

**Figure 4:**
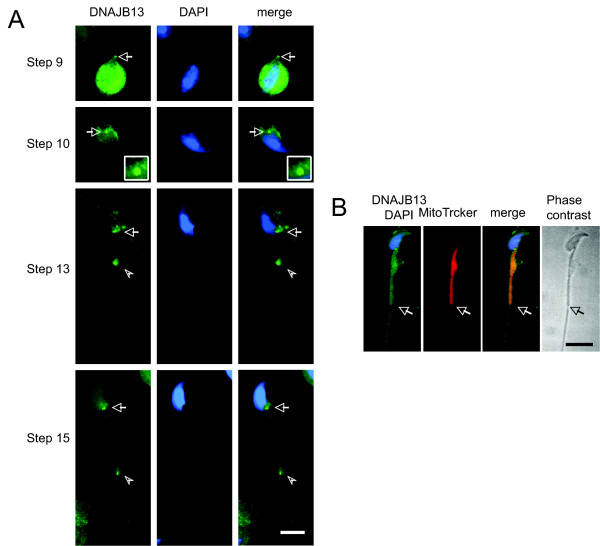
**Localization of DNAJB13 in *Sept4*-null mouse spermatids**. (A) As in wild type spermatids, the annulus-like DNAJB13 staining (green, arrows) was able to be detected at the neck region of spermatids from step 9 to early step 15, but the signal intensity was low and did not change obviously compared with that of the wild type spermatids. Also as in wild type spermatids, the dot (arrowheads) was present and migrated towards the midpiece-principal piece junction. (B) In middle step 15 spermatids, DNAJB13 was undetectable at the midpiece-principal piece junction, which was easily distinguished by staining the mitochondrial sheath with MitoTracker (red). The structural defect of the spermatid caused by the absence of annulus is clearly seen in the phase contrast image. The sperm nuclei were stained with DAPI (blue). Arrows indicated the position where the annulus should be in wild type spermatids. The scale bars represent 10 μm.

To further study the relationship between DNAJB13 and the annulus, we examined the localization of DNAJB13 in *Sept4*-null mouse spermatids. Surprisingly we found that DNAJB13 was still present at the neck region of the flagellum, displaying either a ring-like or two-dot pattern from step 9 to early step 15 (Figure 4A). However, the intensity of the annulus-like DNAJB13 staining in *Sept4*-null spermatids was consistently low and steady, compared to that in wild type spermatids (Figures 3b–f). Perhaps due to lack of the annulus in the *Sept4*-null spermatids, the annulus-like DNAJB13 staining disappeared soon and was undetectable in middle step 15 of the *Sept4*-null spermatids (Figure 4B). These data indicate that the annulus-like DNAJB13 appears prior to formation of the septin-based annulus, and further that it may assist in assembling the septin-based annulus.

As in wild type, the DNAJB13-positive dot was able to be detected on flagella of the *Sept4*-null spermatids, and also migrated towards the midpiece-principal piece junction (Figure 4A). Therefore, its presence and migration are likely to be independent of annulus. Since the mitochondrial sheath is still formed in *Sept4*-null spermatids [8,9], the dot may play a role in mitochondrial sheath orientation in the absence of annulus.

### In vitro DNAJB13 co-localized and interacted with SEPT4

In an effort to determine whether *DNAJB13 *and *SEPT4 *interact in vivo, we first made the testicular lysate. However, the great majority of testicular *SEPT4 *was detergent-insoluble (data not shown). We then turned to an in vitro assay by constructing plasmids expressing GFP-tagged DNAJB13 and FLAG-tagged SEPT4 respectively, co-transfecting them into HeLa S3 cells, and then performing both immunofluorescence and co-immunoprecipitation (Co-IP) analyses.

**Figure 5:**
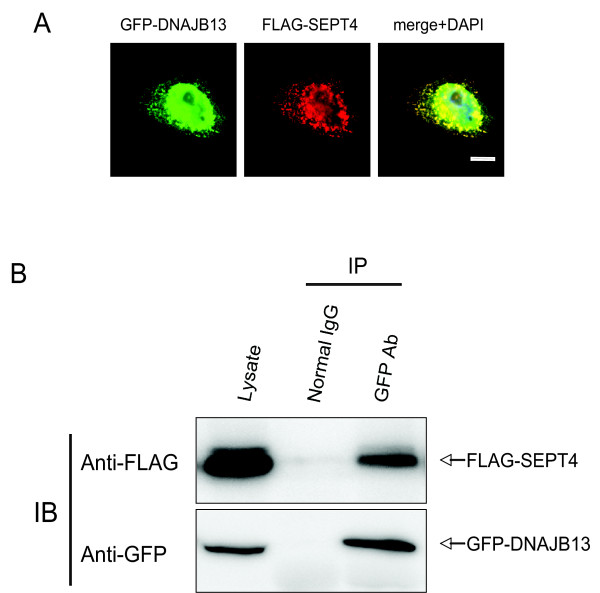
**In vitro studies of DNAJB13 and SEPT4**. (A) GFP-DNAJB13 (green, upper panel) was co-localized with FLAG-SEPT4 (red) in HeLa S3 cells. The cell nuclei were stained with DAPI. The scale bar represents 10 μm. (B) HeLa S3 cells were co-tranfected with plasmids pGFP-*Dnajb13 *and p3xFLAG-*Sept4*. Twenty-four hours later the cells were lysed and subjected to Co-IP with normal rabbit IgG or rabbit anti-GFP antibody. The lysate (input) together with the Co-IP products were then subjected to immunoblotting with mouse anti-FLAG antibody and mouse anti-GFP antibody respectively. IB: immunoblotting.

The immunofluorescence result showed that GFP-DNAJB13 was co-localized with FLAG-SEPT4 in HeLa cells (Figure 5A), consistent with the in vivo findings. The Co-IP result showed that FLAG-SEPT4 could be co-immunoprecipitated with GFP-DNAJB13 by anti-GFP antibody (Figure 5B), indicating that DNAJB13 and SEPT4 interact directly. The same results were also obtained in U2OS and CHO cell lines (data not shown). It is known that HSP40s homodimerize via their C-terminals to capture substrates [20,21]. In fact, RSP16 (radial spoke protein 16), the *Chlamydomonas *orthologue of DNAJB13, can form homodimer [22]. We indeed found that DNAJB13 could homodimerize in HeLa S3 cells (data not shown). Taken together, these data indicate that in vivo DNAJB13 may interact with SEPT4 via its homodimers.

## Conclusion

By performing immunofluorescence and biochemical approaches, we for the first time revealed a spatiotemporal association of DNAJB13, a type II HSP40, with the sperm annulus and elucidated a relationship between DNAJB13 and the annular constituent SEPT4. With closer inspection of the annulus-deficient spermatids from *Sept4*-null mice, we here tentatively propose a model of how DNAJB13 is involved in the assembly and positioning of sperm annulus. In the early stage of sperm flagellum development, DNAJB13 forms an annular anlage, or attaches to a pre-existing annular anlage, and then recruits septins through direct interaction with SEPT4 or SEPT4-containing septin complex to build up a genuine annulus. In the late stage of sperm flagellum development, DNAJB13 may assist the migration of the annulus, and meanwhile forms a dot on the flagellum to direct the annulus toward the midpiece-principal piece junction. As the annulus is positioned to the midpiece-principal piece junction, DNAJB13 disappears gradually with formation and maturation of the mitochondrial sheath.

## Methods

### Animals

All animal procedures were approved by Stockholm-North Animal Ethical Committee or the Animal Care and Use Committee of Kyoto University. C57BL/6N mice were purchased from Charles River, Germany. *Sept4*^+/- ^mice were backcrossed with C57BL/6J for more than 16 generations. *Sept4*^-/- ^mice and the control littermates were generated from *Sept4*^+/- ^mice.

### Plasmid construction

Briefly the full-length open reading frame (ORF) of mouse *Dnajb13 *(accession number NM_153527) were amplified from testis cDNA and cloned in frame into the pEGFP-N1 vector to express GFP-tagged DNAJB13 (pEGFP-*Dnajb13*). The full-length ORF of mouse *Sept4 *(accession number NM_011129) was amplified and cloned in frame into pCMV-3xFLAG-7.1 vector to express FLAG-tagged SEPT4 (p3xFLAG-*Sept4*). For the cloning primers, see table 1.

**Table 1:** Cloning primers.

**Primer name**	**Sequence**
GFP-Dnajb13-forward	5' ATCTCGAGCCATGGGGCTGGATTACT 3'
GFP-Dnajb13-reverse	5' ATAAGCTTGGTCAGCAATGCCTGGCG 3'
FLAG-Sept4-forward	5' AAGTCGACCATTCACTGGGATGG 3'
FLAG-Sept4-reverse	5' ATGGATCCTTAGTGAGTCTCCTTCATCTG 3'

Note: The sequences of flanked restriction endonuclease sites are underlined.

### In vitro studies

HeLa S3 cells were cultured in Dulbecco's modified Eagle's medium (DMEM) supplemented with 10% fetal bovine serum (FBS) in an atmosphere containing 5% CO_2 _at 37°C. Cells were transfected with Lipofectamine 2000 (Invitrogen, Carlsbad, CA) according to the manufacturer's instruction.

For immunofluorescence analysis p3xFLAG-*Sept4 *was co-transfected with pEGFP-*Dnajb13 *into HeLa S3 cells plated onto coverslips. Twenty-four hours later these coverslips were fixed and subjected to immunofluorescence analysis.

For Co-IP analysis, plasmids p3xFLAG-*Sept4 *and pEGFP-*Dnajb13 *were co-transfected into HeLa S3 cells plated in a 10-cm dish. Twenty-four hours later cells were washed once with 1× PBS and then lysed with ELB lysis buffer (50 mM Hepes, pH 7.4, 0.25 M NaCl, 0.5 mM EDTA, 0.1% NP_40) supplemented with 1× protease inhibitor cocktail (Roche) on ice for 30–60 minutes. After lysis the cells were scraped off and transferred into a new eppendorf tube. After centrifuge at 13,000 rpm for 20 minutes at 4°C, a small aliquot of the supernatant was taken as input, and the left was divided into two new eppendorf tubes equally. One tube was added with rabbit anti-GFP antibody (A11122, Invitrogen), and the other was added with normal rabbit IgG. After 2-hour incubation at 4°C with gentle rotation, each tube was added with protein G beads (Upstate) and incubated for another 2 hours. After 4 times wash with lysis buffer, the beads were boiled in 2 × SDS sampling buffer at 95°C for 10 minutes and then centrifuged at 13,000 rpm for 2 minutes to collect the Co-IP products. The input and the Co-IP products were then subjected to immunoblotting analysis.

### Immunofluorescence microscopy

For germ cells, adult mouse testes or epididymes were collected and minced in 1 × PBS to release germ cells. After filtration the cells were attached onto acid-treated, poly-L-lysine-coated coverslips. The coverslips were then fixed in 4% paraformaldehyde (PFA) for 15 minutes and permeabilized in 0.25% Triton X-100 for 10 minutes at room temperature. After blocking in 3% BSA the coverslips were incubated with the primary antibodies for 1–2 hours at room temperature. After rinse the coverslips were then incubated with the secondary antibodies alone or together with MitoTracker Red 580FM (Molecular Probes, Invitrogen) for 30 minutes at room temperature. After rinse and DAPI staining, the coverslips were mounted onto slides with Prolong anti-fade reagents (Molecular Probes, Invitrogen). The primary antibodies used were rabbit anti-DNAJB13 antibody (1:50), rabbit anti-SEPT4 antibody (Santa Cruz, 1:200) and guinea pig anti-SEPT4 antibody (raised against oligopeptide corresponding to residues 466–478 of mouse SEPT4, 1:100). The secondary antibodies used were FITC-conjugated swine anti-rabbit IgG (Dako, 1:100) and Cy3-conjugated donkey anti-guinea pig IgG (Jackson Lab, 1:1,200).

For cultured cells, the coverslips were fixed and permeabilized in 2% PFA + 0.5% Triton X-100 in 1× PBS for 15 minutes at room temperature. The following steps were performed as described above. The primary antibody used was rabbit anti-SEPT4 antibody (Santa Cruz, 1:200). The second antibody was TRITC-conjugated goat anti-rabbit IgG (Santa Cruz, 1:100).

All the slides were examined using a Leica DMRA2 microscope (Leica Corp.), and photographed with Hamamatsu ORCA-HR digital camera (Hamamatsu, Japan) operated by OpenLab software (ImproVision Inc.)

### Immunoblotting

Immunoblotting was performed as described previously [23]. Briefly, protein samples including input and Co-IP products were separated by SDS-polyacrylamide gel electrophoresis (PAGE) and transferred to PVDF membrane. The membrane was blocked with 5% non-fat milk in 1× TBS (blocking buffer) for 1 hour at room temperature and then incubated with primary antibodies in blocking buffer overnight at 4°C. After rinse the membrane was incubated with secondary antibodies in blocking buffer for 1 hour at room temperature. The immunosignal was detected with the LAS-1000 plus image analyzer (FUJIFILM) using ECL kit (Pierce, Rockford, IL). The primary antibodies used were mouse anti-FLAG M2 monoclonal antibody (F-3165, Sigma, 1:20,000), and mouse anti-GFP monoclonal antibody (A11120, Invitrogen, 1:2,000). The secondary antibody was HRP-conjugated goat anti-mouse IgG antibody (DAKO, 1:5,000).

## Authors' contributions

JG participated in the design of the study, carried out all the experiments, drafted and revised the manuscript. MK provided the *Sept4*-null spermatid samples and helped to revise the manuscript. LY participated in its design and coordination, drafted and revised the manuscript. All authors read and approved the final manuscript.

## Supplementary Material

Additional file 1 **Co-localization of DNAJB13 and SEPT4 to the annulus in spermatids**. One spermatid at step 9 and the other at step 11 were doubly stained with antibodies against DNAJB13 (green) and an annulus constituent SEPT4 (red). Immunofluorescence results showed that DNAJB13 was co-localized with SEPT4, indicating a localization of DNAJB13 to the annulus. Insets were enlarged images of the annulus (arrows). The spermatid nuclei were stained with DAPI (blue). The scale bar represents 10 μm. Click here for file

Additional file 2 **Three annulus-staining patterns revealed by the DNAJB13 antibody**. Testicular germ cells were stained with the DNAJB13 antibody. Three typical annulus-staining patterns (arrows) were exhibited by the DNAJB13 antibody: the two-dot pattern (A), the bar-like pattern (B), and the ring-like pattern (C). The sperm nuclei were stained with DAPI (blue). The scale bar represents 10 μm. Click here for file
