# Supplementary figures and images for: Spatiotemporal association of DNAJB13 with the annulus during mouse sperm flagellum development
Source: BMC Dev Biol. 2009 Mar 19;9:23. doi: 10.1186/1471-213X-9-23 (PMC2670831; doi:10.1186/1471-213X-9-23)

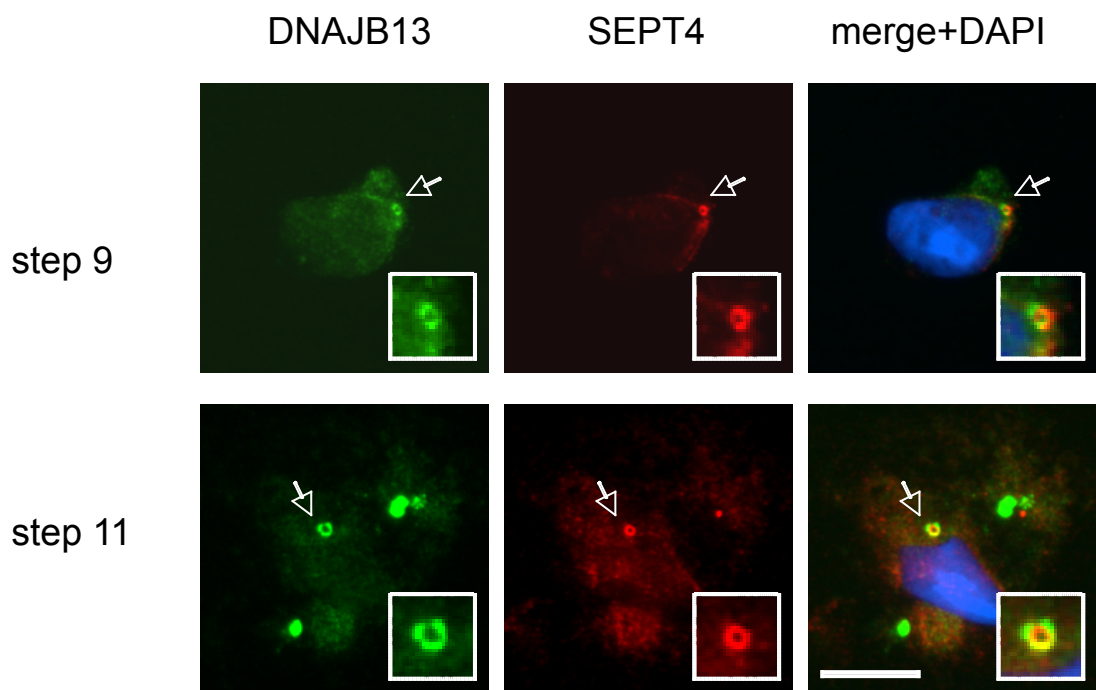

Supplement: Additional file 1 — Co-localization of DNAJB13 and SEPT4 to the annulus in spermatids. One spermatid at step 9 and the other at step 11 were doubly stained with antibodies against DNAJB13 (green) and an annulus constituent SEPT4 (red). Immunofluorescence results showed that DNAJB13 was co-localized with SEPT4, indicating a localization of DNAJB13 to the annulus. Insets were enlarged images of the annulus (arrows). The spermatid nuclei were stained with DAPI (blue). The scale bar represents 10 μm. [file 1471-213X-9-23-S1.pdf]

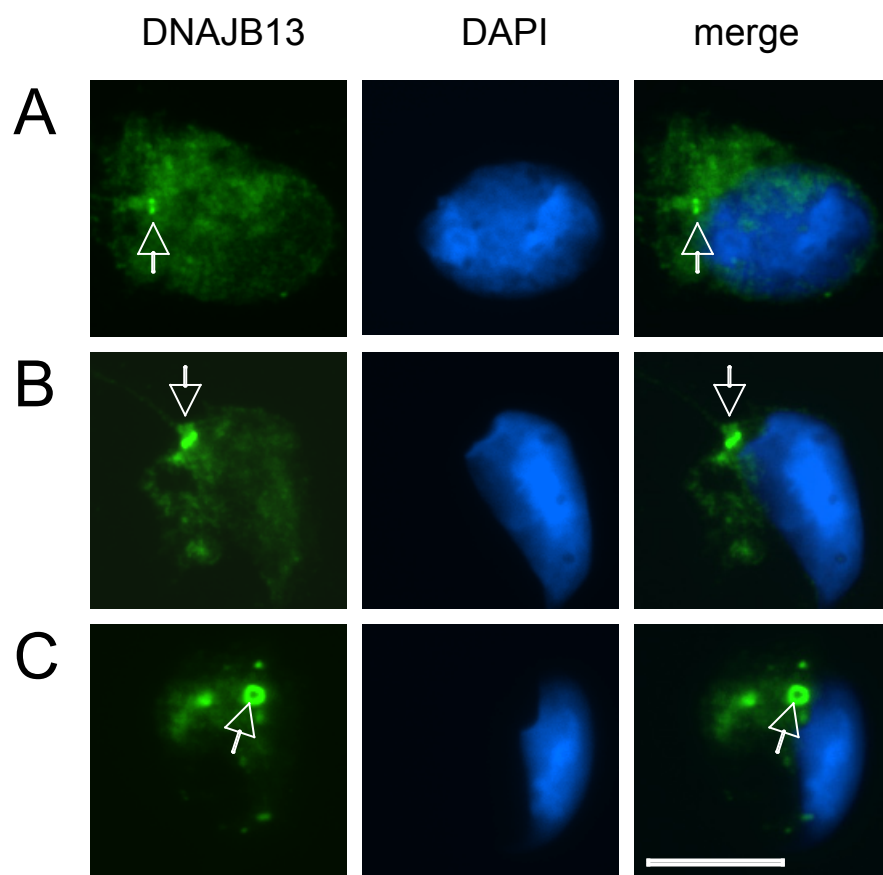

Supplement: Additional file 2 — Three annulus-staining patterns revealed by the DNAJB13 antibody. Testicular germ cells were stained with the DNAJB13 antibody. Three typical annulus-staining patterns (arrows) were exhibited by the DNAJB13 antibody: the two-dot pattern (A), the bar-like pattern (B), and the ring-like pattern (C). The sperm nuclei were stained with DAPI (blue). The scale bar represents 10 μm. [file 1471-213X-9-23-S2.pdf]
